# Physicochemical and Biological Insights Into the Molecular Interactions Between Extracellular DNA and Exopolysaccharides in *Myxococcus xanthus* Biofilms

**DOI:** 10.3389/fmicb.2022.861865

**Published:** 2022-04-22

**Authors:** Yan Wang, Tingyi Li, Weiwei Xue, Yue Zheng, Yipeng Wang, Ning Zhang, Yue Zhao, Jing Wang, Yuezhong Li, Chuandong Wang, Wei Hu

**Affiliations:** ^1^State Key Laboratory of Microbial Technology, Microbial Technology Institute, Shandong University, Qingdao, China; ^2^College of Pharmaceutical Science, Shandong University of Traditional Chinese Medicine, Jinan, China

**Keywords:** extracellular DNA, exopolysaccharides, extracellular matrix, biofilm, macromolecular interactions, *Myxococcus xanthus*, antimicrobial resistance

## Abstract

Extracellular DNA (eDNA) is a critical component in the extracellular matrix (ECM) of bacterial biofilms, while little is known about the mechanisms underlying how eDNA integrates into the ECM through potential macromolecular interactions. *Myxococcus xanthus* biofilm was employed as a suitable model for the investigation due to the co-distribution of eDNA and exopolysaccharides (EPS) owing to their direct interactions in the ECM. DNA is able to combine with *M. xanthus* EPS to form a macromolecular conjugate, which is dominated by the electrostatic forces participating in the polymer-polymer interactions. Without intercalation binding, DNA-EPS interactions exhibit a certain degree of reversibility. Acting as a strong extracellular framework during biofilm formation process, the eDNA-EPS complex not only facilitates the initial cell adhesion and subsequent establishment of ECM architecture, but also renders cells within biofilms stress resistances that are relevant to the survival of *M. xanthus* in some hostile environments. Furthermore, the EPS protects the conjugated DNA from the degradation by nucleic acid hydrolases, which leads to the continuous and stable existence of eDNA in the native ECM of *M. xanthus* biofilms. These results will shed light on developing prevention and treatment strategies against biofilm-related risks.

## Introduction

In both natural and artificial ecosystems, bacterial cells can aggregate and embed in a self-produced macromolecular extracellular matrix (ECM) to form highly organized and structured biofilms, which adhere to the biotic and abiotic surfaces or suspend in the fluid as flocs (Flemming et al., 2016; van Wolferen et al., 2018). The lifestyle of bacterial biofilms differs remarkably from their free-living style represented by the planktonic growth cells, in terms of the population characteristics of internal cells, physical and chemical properties of ECM, and the intercellular interactions (Penesyan et al., 2021). As a self-protective survival strategy developed by prokaryotes in responding to environmental stresses (i.e., predator attack, chemical treatment, nutrient limitation, oxidative stress, hypoxia and drought, etc.; de la Fuente-Nunez et al., 2013; Gambino and Cappitelli, 2016; Gupta et al., 2016), biofilm is not only essential for the survival of bacteria under hostile conditions but also has a wide range of impacts on human life. Medical device-associated infections (Arciola et al., 2018), bacterial contaminations during food processing and storage (Galie et al., 2018), and biofouling in industrial production (Davidson et al., 2016) mainly originate from the failure of bacterial biofilm eradication. Although our increasing understanding of biofilms is rapidly changing the strategies, the control of biofilm formation and the treatment of existing biofilm are still tenuous with few options (Koo et al., 2017), and the ECM is always considered one of the most promising targets to tackle the challenges (Karygianni et al., 2020).

As a physical framework to maintain the biofilm architecture, ECM is normally a mixture of exopolysaccharides (EPS), proteins, nucleic acids, and other components, which provides diverse benefits to the cells within the biofilm, e.g., acting as a molecular glue to facilitate cell adhesion, conferring protections from different stresses, maintaining biofilm structural integrity, and allowing the establishment of nutrient and waste product gradients (Limoli et al., 2015; Karygianni et al., 2020). Moreover, there is some evidence to indicate that ECM also participates in cell-cell communications, cell migration, and genetic exchange either being freely shared with other species or being exclusive to the siblings (Dragos and Kovacs, 2017). In many bacterial biofilms, EPS and proteins are demonstrated as the key components for ECM to fulfill the functions, while extracellular DNA (eDNA) has attracted more attention owing to its biological importance (Montanaro et al., 2011; Panlilio and Rice, 2021). After the exposure to DNase I, the dissipation of *Pseudomonas aeruginosa* young biofilms suggests that eDNA acts as a structural building block in the early events of its biofilm formation (Whitchurch et al., 2002). Ever since then, the eDNA has been elucidated to be crucial in maintaining the structural integrity of ECM and in promoting bacterial biofilm development (Okshevsky et al., 2015; Panlilio and Rice, 2021). While intensive research has focused on defining the functions of eDNA (Das et al., 2013), the mechanisms underlying that eDNA integrates into the ECM network through potential macromolecular interactions remain elusive, which is critical not only to deeply understand the bacterial biofilm establishments but also to prevent and eliminate biofilm-related risks.

*Myxococcus xanthus* is one of the fascinating Gram-negative bacterial groups that have made the successful transition from unicellular to multicellular life (Cao et al., 2015). It has emerged as a versatile model organism in biofilm research due to its complicated lifestyles, including a nondevelopmental biofilm and a highly organized developmental biofilm (*a.k.a.* fruiting body; Jelsbak and Sogaard-Andersen, 2000; Bretl and Kirby, 2016). Within the ECM of *M. xanthus* biofilms, EPS are found to form a dense reticular network surrounding cells, connect neighboring cells to each other as well as to the surface, and constitute the basic skeleton structure of the ECM (Merroun et al., 2003; Hu et al., 2013). It has been suggested that the isolated *M. xanthus* EPS are composed of at least nine different monosaccharides, including galactose, glucosamine, glucose, rhamnose, xylose, arabinose, mannose, N-acetylglucosamine, and N-acetylmannosamine (Behmlander and Dworkin, 1994; Gibiansky et al., 2013), while the exact structure of its EPS is still unknown (Whitfield et al., 2015). According to our previous studies, both nondevelopmental and developmental biofilms formed by *M. xanthus* have been found to contain an extensive amount of eDNA (Wang et al., 2011; Hu et al., 2012a). Most intriguingly, the eDNA is shown to be closely intertwined with the EPS network and followed the same structural pattern with the EPS, which led to the obviously colocalized distributions of eDNA and EPS in *M. xanthus* biofilm ECM (Hu et al., 2012a). These findings strongly indicate the existence of macromolecular interactions between eDNA and *M. xanthus* EPS, which may drive the formation of integrated and organized ECM structures. In this study, we sought to investigate the physicochemical mechanisms and biological functions of eDNA-EPS interactions in *M. xanthus* biofilms.

## Materials and Methods

### Bacterial Strains and Cultural Conditions

*Myxococcus xanthus* DK1622 (wild type; Kaiser, 1979), DK10547 (Welch and Kaiser, 2001), and SW504 (Δ*difA*; Yang et al., 1998) cells were routinely cultured on CTT agar (Hodgkin and Kaiser, 1977) at 30°C. The nondevelopmental and developmental biofilms were prepared as previously described (Hu et al., 2012a). Briefly, the exponential cells were collected, washed three times with MOPS buffer (10 mM Mops, 4 mM MgSO_4_, pH 7.6), and resuspended in MOPS buffer to a final concentration of 5 × 10^8^ cells/ml. After the incubation in eight-well chambered coverslips (Lab-Tek II Chamber Slide System, Nalge Nunc, United States) for 24 h, the nondevelopmental biofilm was observed on the bottom of the well. To grow the developmental biofilm, *M. xanthus* cells were diluted to 2.5 × 10^7^ cells/ml in CTT medium and incubated at 30°C for 24 h. Subsequently, the medium was removed and the well was replenished with the same volume of MMC buffer (10 mM MOPS, 4 mM MgSO_4_, 2 mM CaCl_2_, pH 7.6). After the incubation at 30°C for 24 h, the initial developmental biofilm was obtained, and the mature developmental biofilm was observed after 48 h of incubation. If needed, a total of 200 μg/ml DNase I (Sangon Biotech, China) was added to the inoculum of *M. xanthus* and the medium to remove any eDNA during the growth of biofilms.

### Confocal Laser Scanning Microscopy

The presence of EPS, eDNA/dead cells, viable cells, and cellular membrane was labeled by Alexa 350-conjugated Wheat Germ Agglutinin (WGA, excitation/emission 346 nm/442 nm), SYTOX orange (excitation/emission 547 nm/570 nm), STYO 9 (excitation/emission 480 nm/500 nm), and FM 4–64 (excitation/emission 515 nm/640 nm), respectively. All the fluorescent dyes were purchased from Thermo Fisher Scientific (United States). The images of *M. xanthus* biofilms were acquired on an LSM 700 confocal laser scanning microscopy (CLSM; Zeiss, Germany) using a 60× oil immersion objective or a 40× objective lens. The CLSM images were captured by ZEN software (Zeiss, Germany) and exported using the ImageJ software (Rasband, 1997–2018). The statistical analysis was performed using the colocalization colormap (Jaskolski et al., 2005) and JACoP (Bolte and Cordelieres, 2006) plugins of ImageJ. The Pearson’s correlation coefficient (PCC), overlap coefficients M1 and M2, and an intensity correlation quotient (ICQ) were calculated as previously described (Li et al., 2004).

### Isolation of *Myxococcus xanthus* Chromosomal DNA and EPS

The chromosomal DNA of *M. xanthus* DK1622 was extracted as previously described (Avery and Kaiser, 1983). The protein and nucleic acid-free insoluble EPS (i-EPS) of DK1622 cells were isolated following the standard procedure (Chang and Dworkin, 1994; Hu et al., 2012a), and the soluble EPS (s-EPS) was purified as previously described (Gibiansky et al., 2013) with minor modifications. The sample incubation at 37°C with 200 μg/ml DNase I and RNase (Sangon Biotech, China) were carried out for 24 h to remove the DNA and RNA contaminations from EPS. The Sevag assay (Pan et al., 2019) was employed to remove the remaining proteins. The Bradford Protein Quantification Kit (Vazyme Biotech Co., ltd, China) was used to detect residual protein. The carbohydrate content of purified EPS was determined by the anthrone assay (Roe and Dailey, 1966).

### DNA Precipitation by i-EPS and *Myxococcus xanthus* Cells

The chromosomal DNA was dissolved to a final concentration of 200 μg/ml in 50 mM Tris-HCl buffer (pH 7.5) containing 2 μg/ml propidium iodide (PI). After the addition of 1 mg/ml i-EPS or *M. xanthus* SW504 cells (1 × 10^8^ cells/ml), the mixtures were extensively vortexed, stationarily incubated at 30°C for 120 min, and centrifugated at 12,000 × *g* for 10 min. The fluorescence intensity of the supernatant was measured at an excitation wavelength of 518 nm and an emission wavelength of 620 nm. Triplicate experiments were conducted.

### Isothermal Titration Calorimetry

The calorimetric data of the chromosomal DNA binding to s-EPS were measured at 25°C on a MicroCal PEAQ-ITC system (Malvern Instruments, United Kingdom). The DNA and s-EPS were dissolved in the binding buffer (50 mM Tris-HCl, pH 7.5), respectively. A 300 μl aliquot of DNA solution was placed in the sample cell followed by 19 sequential titrations in 2 μl aliquot injections of s-EPS from a syringe stock solution into the sample cell. The injection interval was 150 s, and the agitation speed was set as 750 rpm. The control titration of s-EPS into the binding buffer without DNA was performed to measure the heat of mixings and dilutions, which was subtracted from the titration results with the presence of DNA. The raw data was analyzed using MicroCal PEAQ-ITC Analysis Software (Ver 1.1.01262). The data were corrected by deducting the equivalent dilution heats of control titration. The integration of the area under each peak and the deduction of the heat of dilution give the thermogram of the molecular interaction between s-EPS and DNA.

### Transmission Electron Microscopy and Scanning Electron Microscopy

Chromosomal DNA solution (10 mg/L) and EPS suspension (10 mg/L) were prepared in Tris-HCl buffer (50 mM) at pH 7.6, and DNA-EPS suspension was prepared by mixing EPS and DNA solutions at a ratio of 1:1. For the transmission electron microscopy (TEM) observation, 2 μl of sample suspension was dripped onto a carbon-coated 400-mesh Ni grid (EMS, United States). After deposition for 1 min, excess water was removed by touching the edge of the grid with a filter paper, followed by adding a drop of 0.7% *w*/*v* uranyl acetate onto the grid. After washing with water three times, the grid was evaporated to dryness at room temperature. The TEM images were taken using a FEI Tecnai G2 F20 microscope (Thermo Fisher Scientific, United States) operated at 80 kV electron beam accelerating voltage in the brightfield mode. For the scanning electron microscopy (SEM) observation, the nondevelopmental biofilms were cultured on the glass coverslips (18 × 18 mm, Sangon Biotech, China). The sterilized glass coverslip was placed into the 12-well cell culture plate (NEST Biotechnology, China) and submerged in a growth medium inoculated with *M. xanthus* DK1622 cells, and DNase I was added if needed. After 24 h incubation, the biofilm samples were washed three times with PBS buffer (137 mM NaCl, 2.7 mM KCl, 10 mM Na_2_HPO_4_, 2 mM KH_2_PO_4_, pH 7.2) and subsequently fixed in 2.5% glutaraldehyde solution overnight at 4°C. The samples were prepared using a standard method for electron microscopy examination (Velicer and Yu, 2003), and observed under a QUANTA FEG250 scanning electron microscope (Thermo Fisher Scientific, United States).

### Atomic Force Microscopy

Chromosomal DNA solution (10 mg/L) and EPS suspension (10 mg/L) were prepared in Tris-HCl buffer at pH 7.6, and DNA-EPS suspension was prepared by mixing EPS and DNA solutions at a ratio of 1:1. For the atomic force microscopy (AFM) observation, 10 μl aliquot of chromosomal DNA, EPS, or DNA-EPS mixture was, respectively, deposited on the surface of a clean and freshly cleaved mica disk, and air-dried at room temperature. AFM imaging was performed on the acoustic mode at 1 Hz scanning speed using a Multimode Nanoscope VIII AFM (Bruker AXS, Germany) equipped with an 80 kHz frequency silicon cantilever (NanoAndMore Corp, United States). The particle diameter and porosity were determined by using ImageJ. Briefly, an image was chosen, the threshold was adjusted to select the pore, and the pore turned red while the background remained black. Once the appropriate threshold was set, the program determined the percentage region covered by the pore to calculate the porosity. The particle diameters were measured manually. Approximately 10 microscopic fields of view were taken from each sample, and the widths of 50 individuals were measured to calculate the mean value.

The force–separation curves were used to determine the nano-characteristics of *M. xanthus* biofilms with or without DNase I treatment. Briefly, the force–separation curves were measured using an Si3N2 MSNL cantilever (Veeco Digital Instruments, United States) with experimentally measured spring constants of 0.03 N/m and a 10 nm tip radius. The AFM tip was pressed against the surface of biofilm at room temperature with a force of less than 2 nN and subsequently retracted to determine the force–separation curves. All the force measurements were recorded at a pulling rate of 1 Hz on the contact mode.

### Resonance Light Scattering Spectroscopy

The resonance light scattering (RLS) spectra of the EPS-DNA conjugates were obtained as previously described (Chen et al., 2013) with some modifications. In order to measure the spectra, the i-EPS sample was sonicated at 50 Hz for 1 h or homogenized through 20,000 rpm/min grinding to increase its solubility. The mixture of 100 μg EPS and 10 μg chromosomal DNA was suspended in 1 ml Tris-HCl buffer (pH 7.5). The solution was vortexed thoroughly and kept at room temperature for 10 min. One milliliter of the sample was added into a standard 1 cm path-length fluorescence microcuvette. The RLS spectra were recorded on a F-4600 fluorescence spectrometer (Hitachi Corp., Japan). The excitation and emission spectra were recorded in the range of 200–550 nm with synchronous scanning (*λ*_Ex_ *= λ*_Em_, Δ*λ* = 0 nm). Both the excitation and emission slit widths were kept at 5.0 nm. The chitosan-DNA complex was prepared according to the method from literature (Liu et al., 2005b) and used as a positive control. The pH values of suspension were adjusted from 5.5 to 7 with HCl solution or from 8 to 12.5 with NaOH solution. Different amounts of NaCl were added to the solutions to change the ionic strength for the RLS spectrum measurements.

### Competitive Displacement Assay

The competitive displacement of polysaccharides for the DNA polyelectrolyte complex was conducted as previously described (Liu et al., 2005b) with minor modifications. Briefly, the chromosomal DNA and ethidium bromide (EB) were dissolved in 50 mM Tris-HCl buffer (pH 7.5) at a final concentration of 10 and 1.6 μg/ml, respectively. An aliquot chitosan (a positive control) or EPS solution was titrated into the DNA-EB solution at various concentrations and incubated at room temperature for 30 min. The fluorescence intensity of mixtures was measured on a Hitachi F-4600 fluorescence spectrometer with an excitation wavelength of 510 nm and an emission wavelength of 600 nm (Tripathy et al., 2013).

### Differential Scanning Calorimetry

The differential scanning calorimetry (DSC) experiments were carried out on a VP-Capillary DSC system (GE Healthcare, United States). The chromosomal DNA was dissolved in 50 mM Tris-HCl buffer (pH 7.5) at a final concentration of 300 μg/ml supplemented with 700 μg/ml EPS or 70 μg/ml chitosan, respectively. Samples were degassed and pre-equilibrated at 4°C using a ThermoVac degassing station (GE Healthcare, United States) to minimize the formation of bubbles before loading into the syringe, sample cell, and reference cell. All samples were equilibrated at 20°C for 15 min, followed by heating from 40°C to 100°C at the scan rate of 1°C/min. The DSC thermograms of excess heat capacity versus temperature were analyzed using the Microcal, LLC ITC package for Origin version 7.0 (Origin Lab Corporation, MA).

### Fourier Transform Infrared Spectroscopy

Chromosomal DNA (100 μg) was mixed with 100 μg s-EPS or i-EPS in 1 ml Tris-HCl buffer (50 mM, pH 7.5) and incubated at room temperature for 30 min. For the DNA-i-EPS complex, the pellet was collected by centrifugation at 10,000 rpm for 10 min, followed by triple washing with double distilled water to remove unbound DNA. The s-EPS-DNA and i-EPS-DNA samples were dried to form films in a vacuum at 50°C overnight. The Fourier transform infrared (FTIR) spectra were measured over 2,000–600 cm^−1^ on a Nicolet iS50 spectrophotometer (Thermo Fisher Scientific, United States).

### Circular Dichroism Spectroscopy

The circular dichroism (CD) detections of chromosomal DNA (50 μg/ml) in combination with s-EPS or i-EPS at different concentrations in 50 mM Tris-HCl buffer (pH 7.5) were conducted on a Chirascan V100 spectropolarimeter (Applied Photophysics Ltd., United Kingdom) equipped with a 150 W air-cooled Xe arc lamp as the light source. The CD spectra were collected in a 10 mm length quartz cuvette within a range of 320–200 nm at 25°C. Three scans were averaged per spectrum, and the spectra were corrected by subtracting the background spectrum of buffer solution.

### DNA Degradation by DNases

To investigate the protective effects of EPS on DNA, the free or i-EPS bounded chromosomal DNA was treated with 50 μg/ml of DNase I or DNase II (Sangon Biotech, China) in the reaction buffer (50 mM Tris-HCl, 10 mM MgCl_2_, pH 7.5) at 37°C for 10 min. The residual DNA was extracted from the samples by phenol/chloroform and electrophoresed on 1.0% agarose gel at 120 V for 40 min. The gels were visualized by EB staining.

### Measurement of Specific Viscosity

The intrinsic viscosity of chromosomal DNA, i-EPS, and DNA-i-EPS complex was measured using an Ubbelohde viscometer in a water bath at 25°C. The specific viscosity *η*_sp_ was calculated by the reported equations: *η*_sp_ = (*η*/*η*_0_) − 1 = (*T*/*T*_0_) − 1, where *T*_0_ is the flow time of solvent and *T* is the flow time of tested solution (Song et al., 2009).

### Susceptibility of *Myxococcus xanthus* Biofilms to the Surfactants and Antibiotics

*Myxococcus xanthus* DK1622 nondevelopmental biofilm was cultured in 24-well flat-bottom cell culture plates (NEST Biotechnology, China) for 24 h as described above, and 200 μg/ml DNase I was supplemented if needed. After exposure to 2 ml MOPS buffer containing 0.1% sodium dodecyl sulfate (SDS) or 0.1% cetylpyridinium chloride (CPC) for 4 h, the suspensions were removed and the residual biofilm was rinsed twice with water by pipetting up and down. The remained biofilms were stained with 1 ml of 0.1% crystal violet solution at room temperature for 5 min. After staining, the plates were washed in slowly running tap water and dried. The bound crystal violet of each well was dissolved in 1 ml 30% acetic acid solution, and the optical density was measured at 590 nm using a microplate reader (Infinite M200 PRO, Tecan, Switzerland). The 24-h nondevelopmental biofilms of DK1622 were prepared as described above. Spectinomycin or streptomycin was added to the medium at a final concentration of 256 mg/L for 24 h, and the survived cells within the biofilms were determined by counting the colony forming unit (CFU) on CTT medium (Hu et al., 2012b). All experiments were performed in triplicate.

### Susceptibility of *Myxococcus xanthus* Cells to the Extrinsically Supplied DNA

*Myxococcus xanthus* DK1622 or SW504 cells were inoculated in CTT medium and incubated with 200 rpm shaking at 30°C for 24 h. The cells were collected by centrifugation and were adjusted to the concentration of 5 × 10^8^ cells/ml using CTT medium. Two milliliters of cell suspension were transferred into a 10 ml centrifuge tube containing 2 ml CTT medium supplemented with 0.5% (*w*/*v*) calf thymus DNA (Sigma-Aldrich, United States), followed by shaking at 200 rpm and 30°C for 36 h. At different time points, the cell density was measured by detecting the optical density at 600 nm. The number of living cells was determined by CFU counting.

### Statistical Analysis

Unless indicated otherwise, SPSS 23.0 software (SPSS Inc., United States) was used for statistical analysis in the current study. All experiments were conducted at least in triplicate. Quantitative data were expressed as mean ± standard deviation. The Student’s *t*-test was utilized for statistical contrast.

## Results

### The Colocalization of eDNA and EPS Reveals Their Close Interactions in the ECM of Both Nondevelopmental and Developmental *Myxococcus xanthus* Biofilms

**Figure 1:**
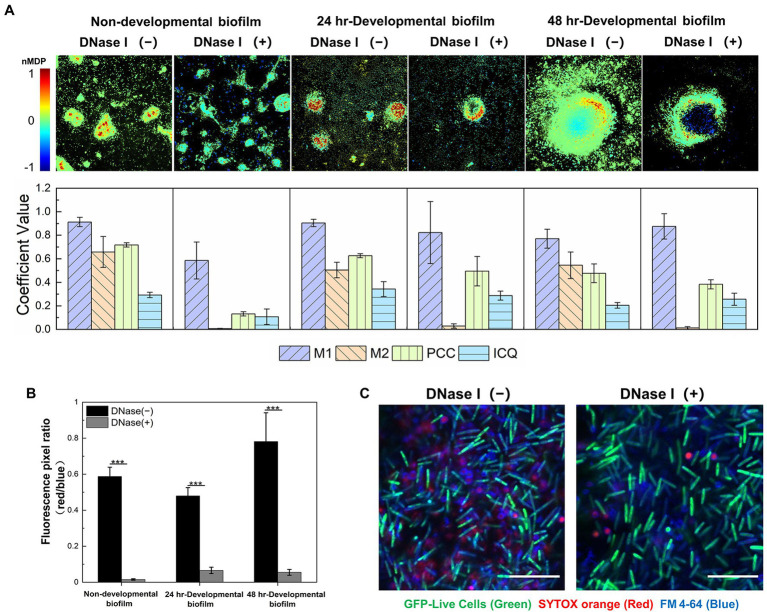
eDNA colocalizes with exopolysaccharides (EPS) in the extracellular matrix (ECM) of both non-developmental and developmental *Myxococcus xanthus* biofilms. **(A)** Quantitative colocalization analysis of the STYOX orange and Alexa 350-conjugated WGA fluorescent signals. The colocalization colormap is shown in the upper panel. The hot color represents a positive correlation, and the cold color represents a negative correlation. Further color divisions within groups are indicated by the color scale bar. In the lower panel, the histogram was generated from the calculated colocalization coefficient (M1 and M2), Pearson’s correlation coefficient (PCC), and an intensity correlation quotient (ICQ; *n* = 5). **(B)** The fluorescence pixel ratio (red/blue) of the samples with/without DNase I treatment. The significance was determined by Student’s *t*-test (*n* = 5; ^***^p < 0.001). **(C)** The non-developmental biofilm (24 h) formed by *Myxococcus xanthus* DK10547 (live cells, GFP-green) was stained with SYTOX orange (eDNA and dead cells, red) and FM 4-64 (cell membrane, blue). Scale bars represent 20 μm.

A nondevelopmental and two developmental (24-h and 48-h) DK1622 biofilms were systematically investigated *in situ* using specific indicator dyes combined with confocal laser scanning microscopy. The viable DK1622 cells with intact membranes were visualized by STYO 9 in green, eDNA and dead cells were stained as red by SYTOX orange, and EPS was revealed in blue using Alexa 350-conjugated WGA. Consistent with the previous observations (Hu et al., 2012a), the red fluorescence presents both dot-like and smeared patterns in nondevelopmental and 24 h-developmental biofilms, while relatively well-organized red structures are revealed in the 48 h-developmental biofilms (Supplementary Figure S1). In the merged panels of Supplementary Figure S1, purple signals are observed as a result of the overlay of blue and red fluorescence. To gain further insight into the colocalization between red and blue signals, a superior visual representation of the spatial description of pixel overlaps and relative intensity was performed by a colormap. As shown in Figure 1A (upper panel), the hot colors represent a positive correlation, while the cold colors represent a negative correlation. By calculating the M1, M2, PCC, and ICQ values, the observed colocalization was further quantified (Figure 1A, lower panel). The M1 coefficient represents the fraction of the red signal colocalized with the blue, and the M2 reflects the fraction of the blue that colocalized with the red. In all native structures (without DNase I), a high level of M1 indicates that a large proportion of the red signals coincides with the blue, while the smaller M2 coefficient suggests a wider distribution of the blue signals compared to that of the red. The PCC and ICQ values describe the correlation of the intensity distribution between the blue and red. The former ranges from −1 (perfect negative correlation) to +1 (perfect positive correlation), and zero means no significant correlation (Dunn et al., 2011). The latter ranges from −0.5 (complete segregation) to 0.5 (complete colocalization), and zero means randomness (Bolte and Cordelieres, 2006). The results show that the red (eDNA) and blue (EPS) signals exhibit a positive colocalization, and most of eDNA coexist with EPS in *M. xanthus* biofilms.

After exposure to DNase I, the red signal in DK1622 biofilms decreases due to the degradation of eDNA, while the green and blue signals that represent live cells and EPS are not affected (Supplementary Figure S1). Statistical analysis confirms a significant reduction of the fluorescence pixel ratio of red versus blue (red/blue) after the addition of DNase I (Figure 1B). It is also observed in Supplementary Figure S1 that only the smeared red signals are disappeared, while most of the dot-like signals remain intact after DNase I treatment. The nucleic acid molecules located in dead cells might be stained as red dots and not susceptible to DNase I digestion. To prove this, the nondevelopmental biofilms formed by *M. xanthus* DK10547 (a *gfp*-expressing derivative of DK1622) were stained with SYTOX orange and membrane-specific dye FM 4-64 (Figure 1C). After DNase I treatments, the detected SYTOX orange signals (red) only exhibit in dotted form encircled by FM 4-64 (blue) labeled membrane, indicating that the smeared red signals came from the eDNA rather than chromosomal DNA in dead or membrane-damaged cells. Furthermore, DNase I remarkably reduces the M2 (*p* < 0.001) but not M1 values in the tested biofilms (Figure 1A), which also supports that most SYTOX orange-stained structures are eDNA. As shown above, eDNA follows a similar pattern of spatial distribution with EPS to participate in the building of *M. xanthus* biofilms, which indicates the potential chemical interactions between these two macromolecules mediate and dominate the construction of a complex ECM network.

### *Myxococcus xanthus* EPS and DNA Interact With Each Other to Form a Macromolecular Conjugate *in vitro*

The purified DNA and EPS from *M. xanthus* DK1622 were employed to investigate the potential DNA-EPS interactions. Due to the interference by EPS, the isolation of a large amount of eDNA from DK1622 biofilms was unsuccessful (data not shown). Considering the possible origin of eDNA and our previous results (Hu et al., 2012a), DK1622 chromosomal DNA was prepared to conduct the subsequent experiments. Both the insoluble EPS (i-EPS) and soluble EPS (s-EPS) were purified from DK1622 cells, and the potential nucleic acid contamination was eliminated by an excessive treatment using nucleic acid hydrolases.

**Figure 2:**
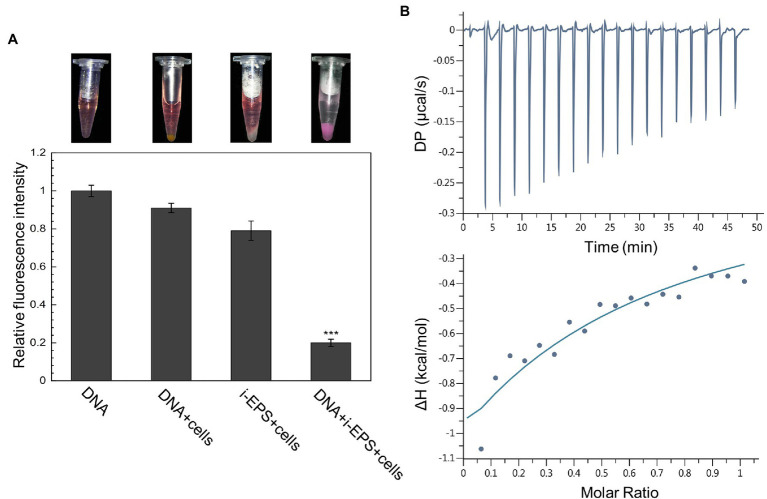
DNA interacts with both i- and s-EPS of *Myxococcus xanthus in vitro*. **(A)** Result of the precipitation assay. The representative photograph of the sample after centrifugation is shown in the upper panel. The fluorescence intensity of the supernatant was measured at an excitation wavelength of 518 nm and an emission wavelength of 620 nm (lower panel; *n* = 3; ^***^p < 0.001). **(B)** Isothermal titration calorimetry (ITC) profile for the titration of s-EPS with DNA. The upper panel represents the raw data for the sequential injections of s-EPS into DNA solution, and the lower panel shows the integrated and dilution-corrected data peak area plotting of the titration data.

According to the different solubility of i-EPS and s-EPS, a precipitation assay and an isothermal titration calorimetry (ITC) analysis were, respectively, employed to investigate their binding abilities with the chromosomal DNA. In the precipitation assay, a red fluorescent dye PI was used to bind and track the DNA molecules. In order to increase the precipitation of i-EPS, the EPS deficient mutant SW504 cells were added into the solution, which was capable of binding with i-EPS (Hu et al., 2011). As shown in Figure 2A, after centrifugation, SW504 cells are not able to pull down DNA from the solution, while they are co-precipitated with i-EPS and DNA, which results in a dramatic decrease of fluorescence signal in the supernatant (*p* < 0.001). The affinity of s-EPS for DNA was determined by an ITC assay, and a representative result is shown in Figure 2B. The observed enthalpy change of DNA and s-EPS interaction is in the range of −0.95 to −0.35 kcal/mol and free energy change (Δ*G*) is −5.43 kcal/mol, which indicates a binding of s-EPS to DNA. The raw corresponding heat evolution curves are caused by the thermal changes during the combination of DNA and s-EPS. The sharp negative peak produced after each individual injection indicates that the process is an obvious exothermic interaction. With the increasing concentration of s-EPS, the amount of released heat decreases gradually, which indicates that the binding sites on DNA are progressively saturated by s-EPS.

**Figure 3:**
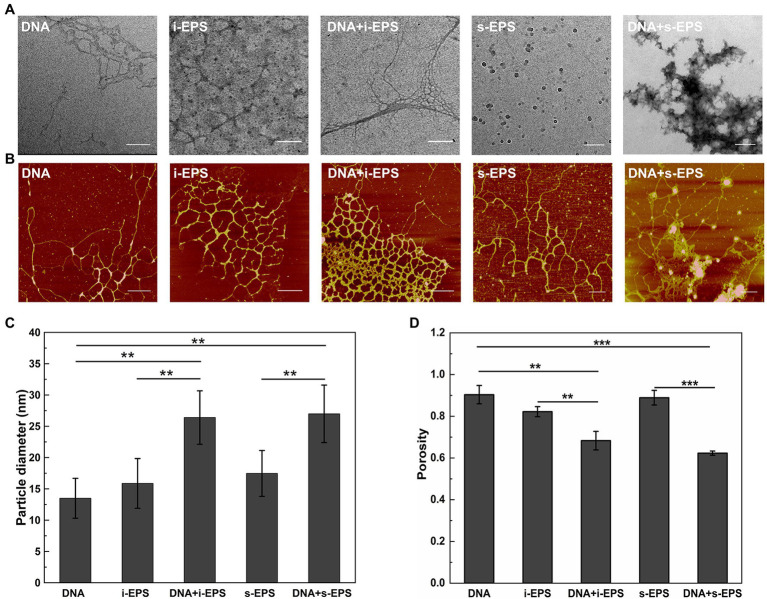
Visualization of the DNA, exopolysaccharides (EPS), and DNA-EPS complex. Representative TEM **(A)** and atomic force microscopy (AFM; **B**) observations of *Myxococcus xanthus* chromosomal DNA (DNA), insoluble EPS (i-EPS), DNA-insoluble EPS complex (DNA + i-EPS), soluble EPS (s-EPS), and DNA-soluble EPS complex (DNA + s-EPS). Scale bars represent 200 nm in **(A)** and 500 nm in **(B)**. The particle diameter of the aggregates **(C)** and the porosity of the molecules **(D)** observed by atomic force microscopy (AFM) were measured by ImageJ software (*n* = 20; ^**^p < 0.01; ^***^p < 0.001).

Ultrastructure of EPS-DNA conjugates was revealed by transmission electron microscopy (Figure 3A). s-EPS forms spherical nano-aggregates with an average radius of 16.80 ± 4.32 nm (*n* = 50), and i-EPS forms both spherical and short rope-shaped aggregates. The rope structures are fairly homogeneous in diameter from 11.03 to 39.16 nm, while variates in length from 85.33 to 1,067.21 nm (*n* = 50). In the DNA + s-EPS sample, smeared patch-shaped aggregates are observed, which is apparently different from the observation on DNA or s-EPS sample. In the DNA + i-EPS sample, strand-like structures are observed, which is different from the i-EPS sample while similar to the shape of DNA. However, the diameter of the DNA + i-EPS strand (20 ± 9.71 nm, *n* = 50) is significantly larger than that of the DNA strand (9.88 ± 3.86 nm, *n* = 50; *p* < 0.001). Interestingly, the DNA + i-EPS structures observed in our study partially resembles the ultrastructure of *M. xanthus* ECM (named as fibrils at that time) previously revealed using scanning electron microscopy (Behmlander and Dworkin, 1994), which suggests that the self-building of ECM network by *M. xanthus* cells follows the similar construction rules of DNA-EPS conjugation *in vitro*.

Due to the fixation process of sample preparation, one question that emerged from the electron microscopy of the conjugates was whether the structures observed by TEM accurately reflected the nature of the DNA-EPS complex. Therefore, the samples were further visualized using atomic force microscopy. As shown in Figure 3B, the individual DNA fragment is filamented, and the individual i-EPS or s-EPS appear as reticular structures. These observations are consistent with the previous findings that many polysaccharides exist as a network structure in an aqueous environment while as a clump or patch-like structure after dehydration (Reese and Guggenheim, 2007; Robic et al., 2009). In the DNA + s-EPS sample, not only the strands become much thicker than those of the DNA or s-EPS sample (Figure 3C), but also some clump-shaped aggregates are observed (Figure 3B). In the DNA + i-EPS sample, more dense reticular structures (Figure 3B) formed by thicker strands (Figure 3C) are revealed. The porosity of the AFM ultrastructure was quantified using the contrast analysis of ImageJ software. The compactness of the DNA-EPS complex structures is significantly higher than that of DNA or EPS samples (Figure 3D), indicating that the conjugation of DNA and EPS results in the formation of larger aggregates. These results demonstrate that *M. xanthus* EPS and DNA interact with each other to form a macromolecular conjugate *in vitro.*

### Electrostatic Force Plays an Essential Role in the DNA-EPS Interactions

**Figure 4:**
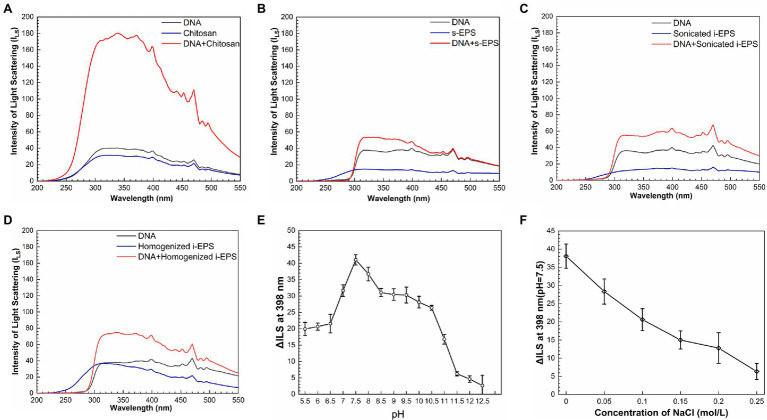
Resonance light-scattering analysis of the interaction between DNA and exopolysaccharides (EPS). The light scattering spectra of chitosan **(A)**, s-EPS **(B)**, sonicated i-EPS **(C)**, homogenized i-EPS **(D)**, and their complex with DNA were detected by fluorescence spectrometer with synchronous scanning in the range of 200–550 nm. The effects of different pH **(E)** and NaCl concentrations **(F)** on the changed value of light scattering spectra intensity (ΔI_LS_) of DNA-homogenized i-EPS complex were measured at 398 nm (*n* = 3). I_LS_, the intensity of light scattering.

To reveal the chemical nature of DNA-EPS interactions, the resonance light scattering spectra of chromosomal DNA, EPS, and DNA-EPS conjugates were measured. As previously reported (Murphy, 1997; Liu et al., 2005a), when two macromolecules bind as a complex, the light scattering intensity of the formed larger particles or aggregates will theoretically increase and positively correlate with the particle size. Chitosan was used as a positive control in the experiment due to its great ability to form conjugates with DNA molecules (Liu et al., 2005b; Bravo-Anaya et al., 2016). As expected, the light scattering intensity of the DNA-chitosan complex is remarkably higher than that of DNA or chitosan (Figure 4A). Similarly, all EPS samples show weak light scattering signals over the wavelength range of 200–550 nm. When DNA is mixed with s-EPS or the pre-treated i-EPS, the light scattering intensity is enhanced, indicating the interaction between DNA and EPS molecules (Figures 4B–D). There are three types of weak forces involved in the interactions among macromolecules within bacterial ECM, i.e., dispersion forces, electrostatic interactions, and hydrogen bonds (Mayer et al., 1999). A variety of studies have well demonstrated that the DNA is a polyanion and forms complex with positively charged chitosan through electrostatic interactions in the natural environment (Agudelo et al., 2016; Bravo-Anaya et al., 2016). Similar to the N-acetyl-glucosamine and glucosamine monomer units composed in the chitosan (Cao et al., 2006), the glucosamine identified in the *M. xanthus* EPS (Behmlander and Dworkin, 1994) probably contributes the cationic moieties of EPS to interact with the negatively charged DNA. To test this hypothesis, the difference between the light scattering intensity at 398 nm (ΔI_LS_) of homogenized i-EPS sample and DNA + homogenized i-EPS complex was measured in the solutions with different pH or ionic strength. As shown in Figure 4E, the light scattering intensity of the DNA-EPS complex is changed according to the pH of the solution and reaches the maximum pH of 7.5. Under extremely high or low pH, the decreased ΔI_LS_ value possibly results from the disturbance of the DNA-EPS combination. The influence of ionic strength on the formation of the DNA-EPS complex was further studied by observing the change of ΔI_LS_ through the addition of NaCl into the solution. With the increased concentration of NaCl, ΔI_LS_ value decreases linearly (Figure 4F), which is likely due to the addition of sodium ions attenuating the electrostatic interaction between DNA and EPS by competition for phosphate groups on the DNA molecules. These observations correspond to the previous reports that the stability of the chitosan-DNA complex depends on the degree of protonation of chitosan (relating to pH) and the external salt concentration (Bravo-Anaya et al., 2019).

**Figure 5:**
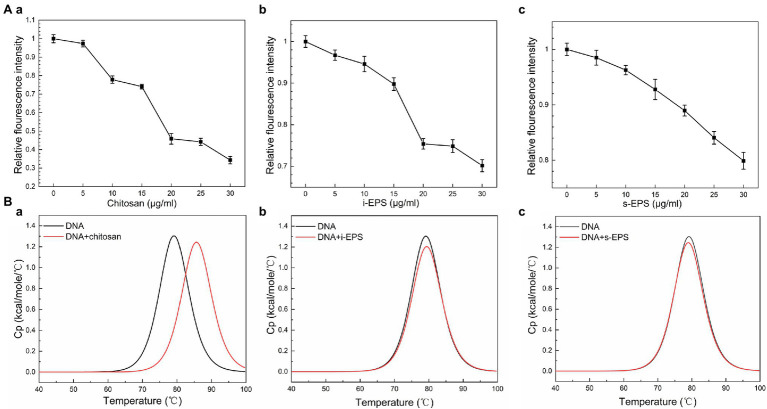
The role of electrostatic force in the DNA-EPS interactions. **(A)** Competitive displacement of EB bond to DNA by chitosan (a), i-EPS (b), and s-EPS (c) at pH 7.5. The fluorescence intensity was monitored using an excitation and emission wavelength of 510 and 600 nm. **(B)** Differential scanning calorimetry (DSC) curves for thermal melting of DNA in the presence of chitosan (a), i-EPS (b), and s-EPS (c). The DSC melting data was generated by subtracting the buffer response and normalized to the concentration of DNA.

As a fluorescent probe, EB remarkably increases its fluorescent intensity after inserting into DNA molecule (Kabir and Kumar, 2014), which is used in the competitive displacement assay to determine the binding affinity of the biomolecules to DNA (Liu et al., 2005b). Consistent with the previous finding (Liu et al., 2005b), the fluorescence intensity of the EB-DNA complex significantly decreases after the addition of chitosan (Figure 5Aa). Similarly, when i-EPS or s-EPS is added, the fluorescence intensity decreases gradually according to the EPS concentration (Figures 5Ab,c), indicating that *M. xanthus* EPS is able to compete for the DNA-binding sites and replace EB from the EB-DNA complex. Moreover, the observed efficiency of EPS (at 30 μg/ml) displacement for EB is only 20–30%, while that of chitosan at 30 μg/ml reaches approximately 70%, suggesting the potential differences between the binding of chitosan-DNA and EPS-DNA. It has been demonstrated that there are two binding modes between EB and DNA, i.e., intercalation binding and electrostatic binding (Vardevanyan et al., 2016). Intercalation occurs due to stacking contact with base pairs, which stabilizes the DNA double helix structure and causes its melting temperature (Tm) to increase by about 5–8°C. Meanwhile, the electrostatic interaction with negatively charged phosphates of DNA will not cause an obvious increase of the Tm (Sun et al., 2008). Therefore, the differential scanning calorimetry assay was used to determine Tm values of DNA-chitosan and DNA-EPS conjugates. The Tm of DNA increases from 77.87°C to 85.71°C with the presence of 70 μg/ml chitosan (Figure 5Ba), while the Tm value remains at approximately 78°C after the addition of 700 μg/ml i-EPS or s-EPS, respectively (Figures 5Bb,c). To further verify this observation, the conformation of DNA molecules in the DNA-EPS conjugate was determined.

### DNA Remains B-Type Conformation in the DNA-EPS Complex

**Figure 6:**
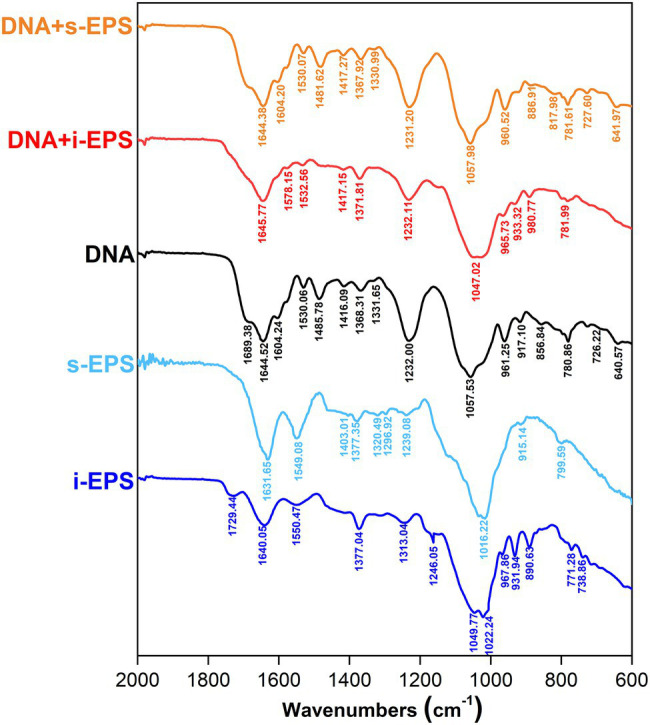
Fourier transform infrared (FTIR) spectra of DNA, exopolysaccharides (EPS), and DNA-EPS complex. The spectrum was collected in the range of 2,000–600 cm^−1^. i-EPS, insoluble EPS; s-EPS, soluble EPS; DNA + i-EPS, complex of DNA and insoluble EPS; DNA + s-EPS, complex of DNA and soluble EPS.

The Fourier transform infrared spectra of chromosomal DNA, i-EPS, s-EPS, and DNA-EPS complexes were determined (Figure 6). In the spectra, the vibrational bands of DNA at 1,604, 1,644, 1,689, and 1,485 cm^−1^ are assigned to adenine (A), thymine (T), guanine (G), and cytosine (C) nitrogenous bases, respectively. The DNA adopts B-type conformation suggested by the phosphoryl ester bond at 1,232 cm^−1^ and the 2′-deoxyribose at 961 cm^−1^. The band observed at 1,631 cm^−1^ (s-EPS) or 1,640 cm^−1^ (i-EPS) is assigned to the NH_2_ scissoring vibration, and the characteristic band of carbonyl asymmetric stretching vibration exhibits at 1,549 cm^−1^ (s-EPS) or 1,550 cm^−1^ (i-EPS). The pyranose ring (890 cm^−1^) of s-EPS or i-EPS is still discernable in the presence of DNA, revealing the formation of the complexes. Compared with the spectrum of DNA, the vibration bands of both DNA + s-EPS and DNA + i-EPS conjugates remain nearly unchanged, suggesting that the B-type conformation of DNA is maintained in the complexes. This conclusion is also supported by the measurement of circular dichroism (CD) spectra (Supplementary Figure S2). DNA appears in a typical B conformation with the base pair perpendicular to the double helix axis. The positive band at ~277 nm is due to base stacking, while the negative band at ~243 nm is due to helicity (Agarwal et al., 2013; Rehman et al., 2015), which are sensitive to the interactions between ligand and DNA (Zhao et al., 2014). As the ratio of EPS to DNA increased, no significant changes are observed both in the positive and negative bands of DNA. Based on these findings, it is concluded that *M. xanthus* EPS binds with DNA to form a complex without changing DNA’s B-type conformation.

### *Myxococcus xanthus* EPS Protects the DNA in the EPS-DNA Complex From the Digestion by DNA Hydrolases

**Figure 7:**
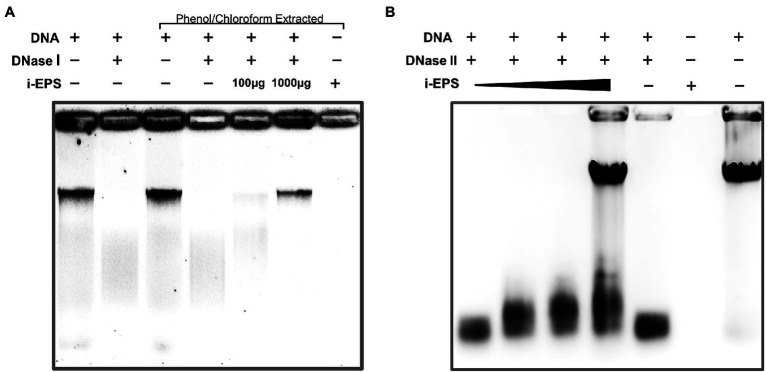
Electrophoretic profile of digested DNA by DNase I **(A)** and DNase II **(B)** in the presence (+) and absence (−) of i-EPS. The DNA was electrophoresed on a 1.0% agarose gel. The gels were stained with EB and photographed under UV light with a gel documentation system. Images of stained DNA fragments were black/white inverted for a better observation.

As shown above, there is a considerable amount of eDNA stably presented in *M. xanthus* biofilms, while *M. xanthus*, as a predatory bacterium, secrets a large number of DNA hydrolases extracellularly (Evans et al., 2012). In order to understand this paradox, the DNA degradations by DNases with or without *M. xanthus* i-EPS were determined. After the treatment with DNase I or DNase II, residual DNA was extracted from the i-EPS-DNA complex using phenol/chloroform and detected by gel electrophoresis. As shown in Figure 7A, the chromosomal DNA without i-EPS is completely digested by DNase I (Lane 2 and 4). In the presence of a sufficient amount of i-EPS, the undegraded chromosomal DNA is detected (Lane 6), which is not due to any eDNA contamination in the isolated i-EPS sample (Lane 7), indicating that i-EPS is able to prevent DNA digestion by DNase I in a concentration-dependent manner. Similar results were obtained from the DNase II degradation experiment (Figure 7B). Small DNA fragments resulting from DNase II digestion of chromosomal DNA are revealed in the absence (lane 5) or in the presence of low concentrations of i-EPS (Lanes 1–3). Despite slight degradation, a high concentration (1,000 μg/ml) of i-EPS prevents DNA degradation by DNase II (Lane 4). These results suggest that, in *M. xanthus* EPS-DNA complexes, the protection by EPS confers DNA resistance to the self-produced nucleic acid hydrolases by cells, which leads to the continuous and stable existence of eDNA in the *M. xanthus* biofilm ECM.

### DNA-EPS Complex Increases the Nanomechanical Strength of *Myxococcus xanthus* ECM

**Figure 8:**
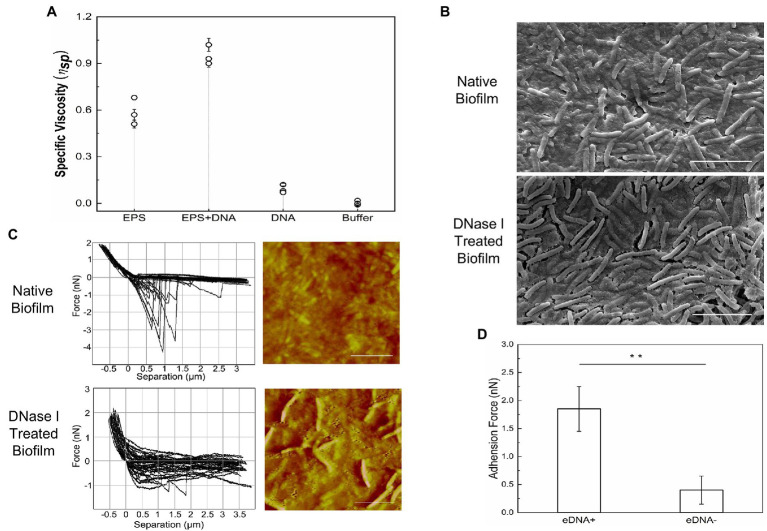
Nanomechanical strength of DNA-EPS complex. **(A)** Specific viscosity of DNA, exopolysaccharides (EPS) and DNA-EPS complex. **(B)** SEM images of native (upper panel) or DNase I treated (lower panel) *Myxococcus xanthus* DK1622 biofilms. Scale bars represent 5 μm. **(C)** Representative force curves and atomic force microscopy (AFM) images of native (upper panel) or DNase I treated (lower panel) *Myxococcus xanthus* DK1622 biofilms. Scale bars represent 5 μm. **(D)** The average adhesion force between extracellular matrix (ECM) with (+) or without (−) eDNA and the tip of AFM cantilever. ^**^p < 0.01.

It is necessary for ECM to reach a certain viscosity and cohesiveness during the initial process of biofilm formation, which is essential to fulfilling its functions in cell adhesion and matrix formation (Verstraeten et al., 2008). The specific viscosity of chromosomal DNA, i-EPS, and DNA-i-EPS complex was measured, respectively. As shown in Figure 8A, *M. xanthus* i-EPS produces a relatively high-viscosity aqueous suspension at the tested concentration, and DNA-i-EPS conjugate exhibits significantly higher specific viscosity (*p* < 0.001), which might be due to the complex molecular interactions between DNA and i-EPS. Under scanning electron microscopy (SEM; Figure 8B), DK1622 cells in the native nondevelopmental biofilm are surrounded by the dense ECM. After the removal of eDNA by DNase I, no significant changes in the morphology of DK1622 cells are revealed, while more cells are exposed out of a slightly damaged ECM. This observation is confirmed by the AFM images (Figure 8C, right panels). Therefore, the nanomechanical properties of the two biofilms ECM were further investigated by determination of force–separation curves using the AFM. As shown in Figure 8C (left panels), compared with the DNase I-treated ECM, more adhesion events arising from the breakage are observed between the tip of cantilever and native ECM. The adhesive events of the native sample usually stop when the tip retraction length reaches approximately 1 μm, while the DNase I-treated sample shows an extended retraction length of approximately 2–4 μm (Figure 8C). Meanwhile, the average adhesion force between the native biofilm and the tip is significantly higher than that of the DNase I-treated biofilm (Figure 8D).

### The eDNA in ECM Is Critical for the Stress Resistance of *Myxococcus xanthus* Cells

**Figure 9:**
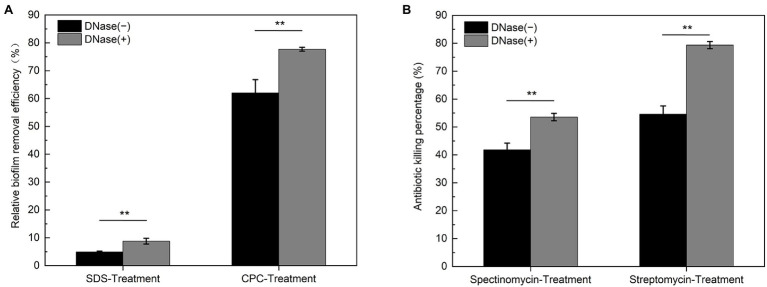
Susceptibility of native and DNase I treated *Myxococcus xanthus* biofilms to the surfactants and antibiotics. The native (DNase I−) and DNase I treated (DNase I+) DK1622 non-developmental biofilms were prepared. **(A)** The relative biofilm removal efficiency was quantified by crystal violet stain after SDS or CPC treatment, respectively. **(B)** The killing percentage by streptomycin and spectinomycin to the cells within biofilms was determined by colony forming unit (CFU) counting, respectively. ^**^p < 0.01.

Chemical disruptions are practical approaches to remove and prevent bacterial biofilms attaching to solid surfaces, where the surfactants are commonly employed (Simoes et al., 2005). As shown in Figure 9A, the cationic surfactant cetylpyridinium chloride (CPC) exhibits a higher efficiency (~60%) in the removal of DK1622 nondevelopmental biofilm than the anionic surfactant sodium dodecyl sulfate (SDS, ~5%). Compared with the native biofilm, the CPC and SDS treatments exhibit a more significant removal efficiency in the biofilm without eDNA in the ECM, ranging to 77 and 8%, respectively. This result suggested that the polyanion DNA molecules trapped in the ECM by interaction with EPS play important roles in resisting the treatment of surfactants, especially for cationic chemicals. Furthermore, the susceptibilities of *M. xanthus* biofilms to cationic antibiotics in the presence or absence of DNA were determined. Streptomycin and spectinomycin are cationic aminoglycosides capable of killing planktonic *M. xanthus* DK1622 cells (Magrini et al., 1998) at the concentrations used in the experiment. As shown in Figure 9B, the killing percentage of DK1622 cells in the native biofilm is approximate 42% for spectinomycin and 55% for streptomycin, which becomes more evident in the presence of DNase I, especially for the streptomycin treatment (~80%).

**Figure 10:**
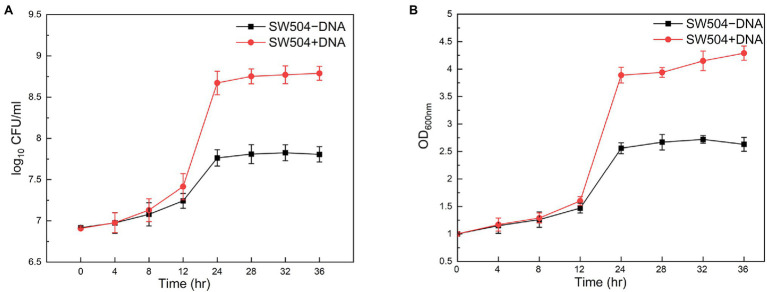
Susceptibility of *Myxococcus xanthus* SW504 cells to the extrinsically supplied DNA. Growth curves of *Myxococcus xanthus* SW504 cells in the CTT medium supplemented with (+) or without (−) 0.5% (*w*/*v*) calf thymus DNA were plotted by measuring the optical density at 600 nm **(A)** and colony forming unit (CFU) counting **(B)**.

Moreover, it has been shown that eDNA at a final concentration of 0.5% (*w*/*v*) definitely inhibits the growth of *P. aeruginosa* planktonic cells (Mulcahy et al., 2008). When 0.5% calf thymus DNA was supplied in the CTT medium, the growth of wild-type *M. xanthus* DK1622 cells is not affected compared with the cells in the medium without DNA supplement (Supplementary Figure S3). This observation can be explained by the fact that EPS on the cell surface conjugates with DNA and prevents its further bactericidal effect. To test the hypothesis, the EPS deficient mutant SW504 cells were cultured in the medium with or without supplied DNA. Unexpectedly, both the cell concentration and live cell number of SW504 slightly increased after incubation for 12 h in the medium with 0.5% DNA (Figure 10). Furthermore, compared to cells in the DNA-free medium, SW504 is able to grow and reach a significantly higher cell concentration in the CTT medium with DNA, which has been observed in some other bacterial species (Mulcahy et al., 2010; Chimileski et al., 2014; Gumpenberger et al., 2016; McDonough et al., 2016). Similar results were obtained when DNA was added in the medium to a higher concentration than 0.5% (data not shown). These results suggest that the EPS deficient *M. xanthus* cells are not only insusceptible to extrinsic DNA but also capable of utilizing the DNA as a nutrient to support their growth.

## Discussion

The focus on the investigation of some model bacteria has revealed that the formation of biofilm is a complex process with multiple factors, and the interactions among ECM components are essential for the adaptive structures in the process of adhesion, maturation and dispersion (Hobley et al., 2015; Payne and Boles, 2016). It is necessary to understand the complexity of macromolecular interactions within biofilms, which will provide a rationale for multi-targeted treatments to either prevent the initiation of biofilms or disrupt mature biofilms (Koo et al., 2017).

Bacteria are able to produce eDNA through different specific mechanisms, e.g., cell autolysis, active secretion, and association with the membrane vesicles (de Aldecoa et al., 2017). Prolific outer-membrane vesicles have been observed in the *M. xanthus* biofilms (Evans et al., 2012), which contain a complex mixture of lipids, carbohydrates, secondary metabolites, and proteins, but not nucleic acids (Berleman et al., 2014; Remis et al., 2014). The coexistence of dead cells and eDNA in the same *M. xanthus* biofilm structure suggests that cell lysis is at least one of the major sources for eDNA production, which is supported by the previous findings that extensive programmed cell death events occur during the starvation-induced *M. xanthus* developmental process (Sogaard-Andersen and Yang, 2008). While abundant eDNA is found to present a well-organized structure similar in appearance to *M. xanthus* EPS organization, the potential molecular interactions between eDNA and EPS are still unknown (Hu et al., 2012a). In this study, the co-distribution of eDNA and EPS in the ECM of *M. xanthus* nondevelopmental and developmental biofilms was proved to be attributed to their direct interactions, which allows eDNA to combine with EPS to form a macromolecular conjugate. Complexing with DNA, *M. xanthus* EPS forms a cohesive, dense, and mechanically strong framework to facilitate the initial cell adhesion and subsequent ECM architecture construction during the biofilm formation process. Due to technical problems in isolation of enough amount of eDNA from *M. xanthus* biofilms, it is reasonable to use DK1622 chromosomal DNA as a substitute for eDNA to conduct the *in vitro* experiments considering the most possible origin of its eDNA. Moreover, small sized commercial salmon sperm DNA has been shown to bind with *M. xanthus* EPS *in vitro* (Hu et al., 2012a), which resembles the observations in current study. It is suggested that the interactions between eDNA and *M. xanthus* EPS are independent of the size and source of DNA. In a multispecies microbial community, this nonspecific EPS-DNA binding facilitates *M. xanthus* cells to absorb the eDNA from other bacteria and environment to grow its own biofilm, which may be one of the reasons that myxobacteria are a ubiquitous group of microorganisms living in very diverse habitats.

Our results demonstrate that, as a potential analogue of chitosan, *M. xanthus* EPS binds to DNA through electrostatic interactions, and the stability of the EPS-DNA complex is significantly influenced by environmental pH and ion concentration. The smaller light scattering intensity of the EPS-DNA conjugate compared with that of the chitosan-DNA complex implies the relatively lower affinity between EPS and DNA, which is supported by the results of EB competitive displacement assay. Consistently, the intercalated interaction is not detected between DNA and EPS by the measurement of Tm values using DSC, while it plays an important role in the combination of chitosan with DNA (Liu et al., 2005b). The unchanged Tm value and B-type conformation of DNA after complexing with EPS demonstrate the lack of insertion mode within their interactions, which is also due to the weaker binding between DNA-EPS. Among the polymer-polymer interactions, the electrostatic forces dominate DNA-EPS complex formation in *M. xanthus* ECM, and the relatively weaker interactions exhibit a certain degree of reversibility. It has been reported that EPS blocks the plasmid transformation in *M. xanthus* (Wang et al., 2011), and the sufficient EPS produced by wild-type cells reduces the local concentration of plasmid DNA to access the cell surface. Our findings explain that *M. xanthus* becomes naturally transformable when the EPS production is dramatically down-regulated or the cells are grown under specific conditions (Wang et al., 2011).

Several studies have shown that DNA could be a nutrition source for bacterial cells (Mulcahy et al., 2010; Chimileski et al., 2014; Gumpenberger et al., 2016; McDonough et al., 2016). As we have shown above, EPS deficient *M. xanthus* cells (like SW504) are capable of utilizing the DNA to support their growth, while the same phenomenon has not been observed in the wild type strain DK1622. As a predatory bacterium, *M. xanthus* can produce a large number of nucleases to hydrolyze the DNA released from the prey for self-growth (Evans et al., 2012), which is contradictory to the fact that abundant eDNA exists in the *M. xanthus* ECM. After elucidating the interactions between EPS and eDNA, it is proposed that *M. xanthus* cells with sufficient EPS are covered by EPS-eDNA complexed ECM, and EPS not only protects eDNA from the nuclease degradation but also prevents the utilizing of eDNA by the cells. Under the conditions of poor nutrition, *M. xanthus* lacks enough energy and materials to synthesize large amounts of EPS, which provides the possibility for wild-type strain to use DNA as nutrition. Indeed, within the starvation induced nondevelopmental biofilm, *M. xanthus* cells with less EPS occasionally disperse from the top of the structure and colonize to a new surface, in that case, eDNA is able to support their subsequent activities. In the developmental biofilms, myxospores are released from the mature fruiting bodies and germinate to produce EPS-free vegetative cells in a favorable environment, where the eDNA is also used as a source to provide nutrition. These results imply that the precisely regulated EPS production is important for *M. xanthus* cells to use eDNA either as a structural component for biofilm establishment or as a nutrient for cell growth.

In *M. xanthus* biofilms, the integrated DNA-EPS matrix confers cells stress resistances against certain surfactant and antibiotic treatments, suggesting that the presence of eDNA and its interaction with EPS is highly relevant to the survival of *M. xanthus* in hostile environments. Consistent with the findings in some pathogenic bacteria, eDNA enhances the resistance of their biofilms to aminoglycoside (Wilton et al., 2016) and glycopeptide antibiotics (Doroshenko et al., 2014). For many established cases, DNase treatment serves important functions in dispersing biofilm and increasing cell susceptibility to biocides and antibiotics by removal of eDNA. Therefore, eDNA is regarded as an promising target for biofilm control through the combination of DNase and drugs therapy, and a better understanding of molecular mechanisms involving eDNA interactions with EPS will help to treat and prevent hospital-derived microbial biofilm infections.

## Conclusion

This study elucidates the physicochemical mechanism and biological functions of the eDNA-EPS interactions in *M. xanthus* biofilms. Under CLSM, abundant eDNA possibly released from the lysed dead cells exhibits a spatial structure similar to the *M. xanthus* EPS organization in the biofilms. This co-distribution of eDNA and EPS is on account of their direct interactions, which allows EPS to combine with eDNA to form a macromolecular conjugate. It is also demonstrated that the electrostatic forces participating in the polymer-polymer interactions dominate the DNA-EPS complex formation in *M. xanthus* ECM. Due to the lack of intercalation by EPS, the binding ability of DNA-EPS is relatively weaker than that of DNA-chitosan, and DNA remains its B-type conformation in DNA-EPS conjugate, which endows a certain degree of reversibility for the complex. Acting as a cohesive, dense, and mechanically strong network, the eDNA-EPS complex facilitates the initial cell adhesion and subsequent ECM architecture establishment, and renders cells within biofilms stress resistances relevant to the survival of *M. xanthus* in some hostile environments, e.g., the presence of surfactants and cationic antibiotics, and nutrient limitation. Furthermore, in the DNA-EPS complex, the protection by EPS confers DNA resistance to the degradations by self-produced nucleic acid hydrolases, which leads to the continuous and stable existence of eDNA in *M. xanthus* ECM. As a potential target for microbial biofilm control, a better understanding of interactions between eDNA and other ECM components will shed light on developing novel prevention and treatment strategies against biofilm-associated risks.

## Data Availability Statement

The original contributions presented in the study are included in the article/**Supplementary Material**, further inquiries can be directed to the corresponding authors.

## Author Contributions

YaW, TL, WX, and YZe performed the experiments. YiW, NZ, and YZa analyzed the experimental results. JW and YL provided resources. YaW and CW wrote the manuscript. CW and WH revised the manuscript. JW, CW, and WH provided financial support. All authors contributed to the article and approved the submitted version.

## Funding

This research was funded by National Key R&D Program of China (2021YFC2101000, to WH), National Natural Science Foundation of China (32070100, to WH), Taishan Industry Leading Talent Program (Tscy20200334, to WH), China Postdoctoral Science Foundation (2020M672048, to CW), Shandong Provincial Natural Science Foundation (ZR2021MC082, to JW, ZR2021QC087, to CW), and Science and Technology Planning Project of Traditional Chinese Medicine of Shandong Province (2019-0027, to JW).

## Conflict of Interest

The authors declare that the research was conducted in the absence of any commercial or financial relationships that could be construed as a potential conflict of interest.

## Publisher’s Note

All claims expressed in this article are solely those of the authors and do not necessarily represent those of their affiliated organizations, or those of the publisher, the editors and the reviewers. Any product that may be evaluated in this article, or claim that may be made by its manufacturer, is not guaranteed or endorsed by the publisher.
