# Supplementary material for: Physicochemical and Biological Insights Into the Molecular Interactions Between Extracellular DNA and Exopolysaccharides in Myxococcus xanthus Biofilms
Source: Front Microbiol. 2022 Apr 22;13:861865. doi: 10.3389/fmicb.2022.861865 (PMC9073016; doi:10.3389/fmicb.2022.861865)
Supplement: Supplementary file 1 [file Data_Sheet_1.pdf]

Supplementary Images

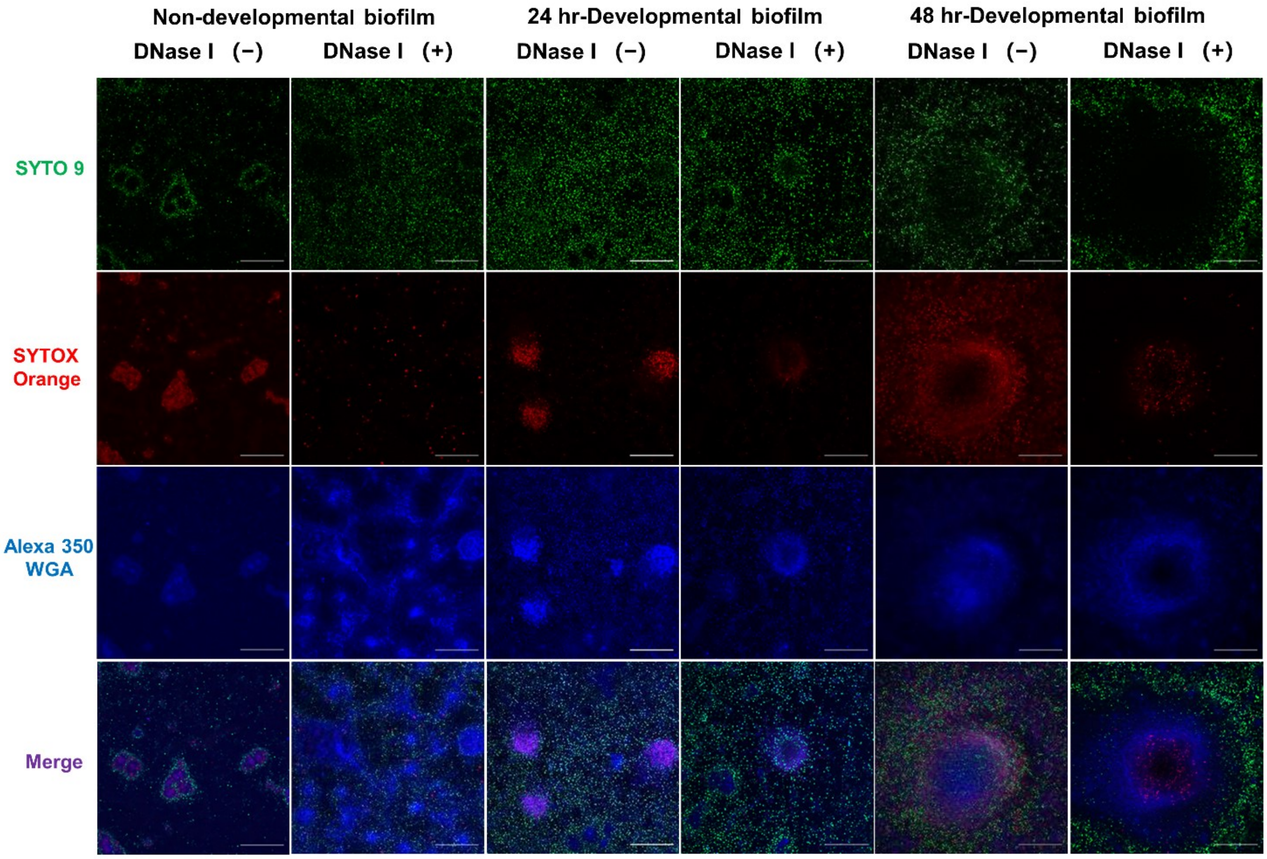

Supplementary Figure S1. The spatial distribution of eDNA, EPS, live and dead cells in *M. xanthus* DK1622 biofilms. The biofilms with or without DNase I were respectively stained with SYTO 9 (living cells, green), STYOX orange (eDNA and dead cells, red), and Alexa 350-conjugated WGA (EPS, blue). All the structures were observed under a confocal laser scanning microscope (CLSM). The merged images of living cells, eDNA, and EPS were shown in the bottom panels. Scale bars represent 50 μm.

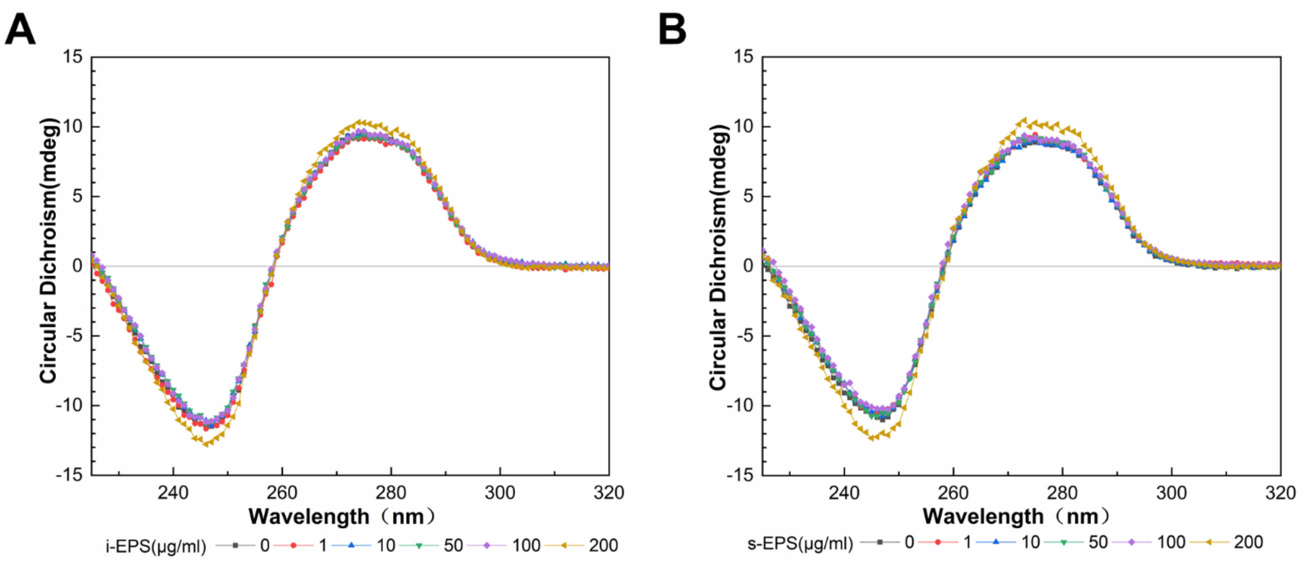

Supplementary Figure S2. CD spectra of DNA in the presence of the different amounts of i-EPS (A) and s-EPS (B). The chromosomal DNA was dissolved in 50 mM Tris-HCl buffer (pH 7.5) at a concentration of 50 μg/ml. The spectra were corrected by subtracting the background spectra of the Tris-HCl buffer.

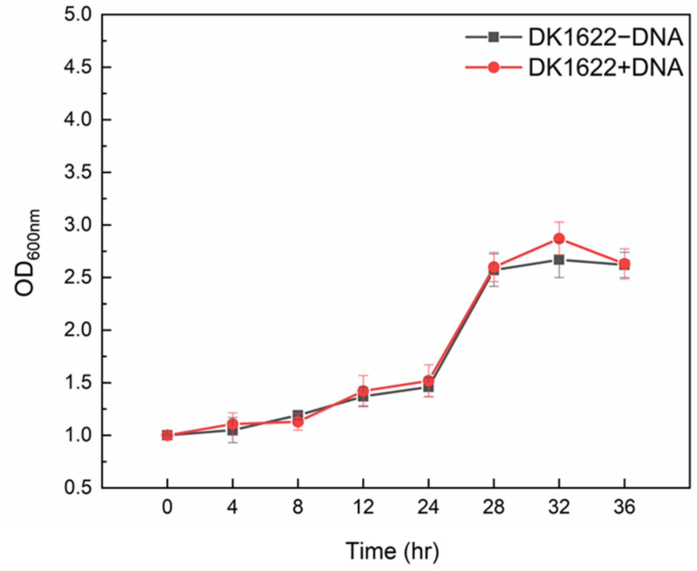

Supplementary Figure S3. Susceptibility of *M. xanthus* DK1622 cells to the extrinsically supplied DNA. Growth curves of *M. xanthus* DK1622 cells in the CTT medium supplemented with (+) or without (-) 0.5% (w/v) calf thymus DNA were plotted by measuring the optical density at 600 nm.
